# MyD88 self-assembles into supramolecular filaments to amplify NF-κB signaling

**DOI:** 10.1016/j.fmre.2025.02.010

**Published:** 2025-02-25

**Authors:** Jia Wang, Xincheng Zhong, Chenyi Liao, Yuchen Zhang, Siqi Shen, Ran Zhang, Guohui Li, Hang Yin

**Affiliations:** a State Key Laboratory of Membrane Biology, School of Pharmaceutical Sciences, Tsinghua-Peking Center for Life Sciences, Key Laboratory of Bioorganic Phosphorous Chemistry and Chemical Biology (Ministry of Education), Tsinghua University, Beijing 100084, China; b Dalian Institute of Chemical Physics, Chinese Academy of Sciences, Dalian 116023, China; c MOE Key Laboratory of Protein Science & Collaborative Innovation Center of Biotherapy, School of Medicine, Tsinghua University, Beijing 100084, China

**Keywords:** Myd88, Filament, Innate immunity, Nf-κb, Assembly

## Abstract

MyD88 is a central player in innate immunity. The molecular mechanism of MyD88 mutation-related diseases largely remains elusive. Here, we report that MyD88 self-assembles into high-order supramolecular structures as a signal amplification machinery. We show that the combination of the death domain (MyD88^DD^) and the intermediate domain (MyD88^ID^) is the minimum unit for the filament formation, which is modulated by the TIR domain (MyD88^TIR^). The loss-of-function mutant L93P disrupts the filaments; the gain-of-function mutant L252P transforms the filaments into speckles. Atomic molecular dynamics simulations demonstrate that the L93P mutation induces conformational changes of MyD88^DD^ by disrupting its hydrophobic core. The L252P mutation causes the unfolding of an α-helix between residues 246–252 and more exposure of the BB-loop. These pathologically relevant mutations alter the MyD88 assembly structure and function, which may contribute to a variety of disease development processes.

## Graphical abstract

**Figure:**
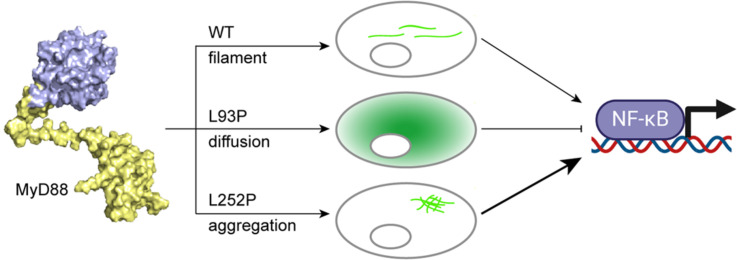

## 1 Introduction

The immune system recognizes pathogens’ invasion by various families of pattern recognition receptors (PRRs). Upon detection of pathogen-associated molecular patterns (PAMPs) or damage-associated molecular patterns (DAMPs), Toll-like receptors (TLRs), one central family of PRRs, rapidly transform into homo- or hetero-dimers and recruit adaptor proteins to initiate the downstream signal transduction [1]. Among the five TLR adaptor proteins, Myeloid differentiation primary response 88 (MyD88) is critical for signal responses of all TLRs except for TLR3 [2]. MyD88 harbors three domains, including a death domain (DD), an intermediate domain (ID) and a Toll/interleukin-1 receptor (TIR) domain [3]. Structurally, the DD of MyD88 (MyD88^DD^) forms myddosome with IRAK4 and IRAK2 by a molar ratio of 6:4:4 [4]. MyD88^DD^ belongs to the death domain superfamily and shares a common six-helix bundle fold [5,6]. The ID of MyD88 (MyD88^ID^) plays a vital role in recruiting IRAK4 and activating downstream NF-κB signaling [7]. This domain is also crucial for the efficient assembly of the myddosome [8]. The TIR domain of MyD88 (MyD88^TIR^) is responsible for sorting and interacting with TIR domain-containing adapter protein (TIRAP; also known as MAL) or TLRs [3,9,10]. Structures of MyD88^TIR^ reveal that MyD88^TIR^ consists of five parallel β-sheets and five α-helices [10,11]. MyD88^TIR^ can form dimers and even oligomers [[12], [13], [14]]. Nonetheless, the overall structure of full-length MyD88 assembly remains unavailable to date.

Dysfunctions of MyD88 are associated with many diseases. MyD88-deficient mice are reported to be highly susceptible to various pathogens [15]. The E52del, L93P and R196C single point natural mutations are found in patients suffering from recurrent pyogenic bacterial infections [15]. MyD88 single nucleotide polymorphisms (SNPs), such as S34Y and R98C, are reported to interfere with myddosome assembly [16]. In patients with activated B-cell-like (ABC) subtype of diffuse large B-cell lymphoma (DLBCL), the L252P (L265P) substitution in MyD88 is the most frequently detected mutation [[17], [18], [19]]. Other mutations, such as V204F (V217F), S206C (S219C), S209R (S222R), M219T (M232T), S230 N (S243 N) and T281P (T294P), are also malignant driving-factors in ABC DLBCL [17]. The L252P, V204F and M219T mutations in the TIR domain have a gain-of-function effect, causing spontaneous activation of NF-κB, JAK-STAT3 and type I interferon signaling. Furthermore, the L252P (L265P) mutation is found in 70%−90% of patients with Waldenstrom macroglobulinemia (WM) [20,21], an incurable, IgM-secreting lymphoplasmacytic lymphoma. MyD88 somatic mutations are also considered as driver mutations for chronic lymphatic leukemia (CLL) [22,23], a slow-growing B cell malignancy. Although plenty of studies have investigated the mechanism of diseases related to MyD88, the detailed molecular basis remains elusive. Upon stimulation of PAMPs or DAMPs, TLRs form dimers, undergo conformation alterations, and recruit MyD88 and IRAK4 to activate the downstream signals. Following the phosphorylation of IRAK4, IRAK1/IRAK2 is involved in forming the large complex named myddosome. The five adaptor proteins in the TLR pathways are MyD88, TIRAP, TIR-domain-containing adaptor protein inducing IFNβ (TRIF; also known as TICAM1), TRIF-related adaptor molecule (TRAM; also known as TICAM2) and sterile α- and armadillo-motif-containing protein 1 (SARM1) [24]. The TIRAP is reported to assemble into filamentous structures [25]. Although MyD88 can form large oligomers with IRAK4 and IRAK2/IRAK1, whether MyD88 has a similar ability to form the filament structure remains ambiguous.

Here, we find that MyD88 forms fibrils both at the cellular level and in the isolated form. MyD88^DD^+MyD88^ID^ (MyD88^DD+ID^) is the minimum unit for MyD88 filament formation. MyD88^TIR^ synergizes in filament growth by regulating MyD88 self-assembly. The L93P mutant completely disrupts the morphology of fibrillar MyD88, but the R196C mutant remains as filaments. Interestingly, other mutations in MyD88^TIR^, including V204F, M219T and L252P, all transform the filaments into speckles. Results from an established assay reveal that MyD88 oligomerization is a prerequisite for activating the NF-κB signaling. The L93P mutation induces conformational changes of MyD88^DD^, leading to failure of fibrillar assembly and blockage of signaling. The L252P mutation, on the other hand, disrupts an α-helix structure between residues 246–252 in MyD88^TIR^, increases the exposure areas of the BB-loop, and results in more robust activation of downstream signals. Changes initiated by the MyD88 mutations can either trigger an inadequate immune response or immune overreaction. In brief, our study brings novel understandings of the MyD88-dependent TLRs pathway, which may facilitate future development of immuno-modulators as potential therapeutics.

## 2 Materials and methods

### 2.1 Cell culture

HEK293T, 293T/17, HeLa, HEK-Blue™ hTLR4 and HEK-Blue™ hTLR5 cells were cultured in DMEM supplemented with 10% fetal bovine serum, 2 mM l-glutamine, 100 µg/ml streptomycin, and 100 U/ml penicillin. Cells were maintained at 37 °C in an incubator with 5% CO_2_. The plasmids were transfected into HEK 293T, HeLa, HEK-Blue™ TLR4 and HEK-Blue™ TLR5 cells using Lipofectamine 3000 according to the manufacturer’s instructions.

### 2.2 Bacterial strain

*Escherichia coli* BL21 cells were used for recombinant proteins expression. Cells were grown in LB medium with the indicated antibiotics and induced with appropriate concentration of IPTG.

### 2.3 Fluorescence imaging

HeLa cells were transfected with indicated plasmids and grown on a coverslip for 20–24 h. Cells were washed twice with PBS, fixed with 4% paraformaldehyde, followed by incubation with DAPI (0.1 μg/ml) in PBS. Images were collected using a Nikon A1R MP confocal microscope or a Nikon super-resolution SIM with a 100× oil-immersion objective. All images were processed with Image J software. Antibodies used include anti-FLAG (MBL, PM020), anti-β-actin (Huaxingbio, HX18201), Tubulin-Tracker Red (Beyotime, C1050).

### 2.4 Generation of cell lines

pLVX-MyD88-GFP, pLVX-MyD88 L93P-GFP, pLVX-MyD88 L252P-GFP plasmids were constructed for lentivirus production. HEK293T cells were transfected with these plasmids plus pMG2.G and psPAX2 and the virus particles were harvested 48 hr after transfection, filtered through a 0.45 μM membrane filter and stored in −80 °C. The HEK-Blue™ hTLR4 and HEK-Blue™ hTLR5 cells were infected by the lentivirus in the presence of 8μg/mL polybrene. After 48 h of culture, transduced cells were selected with 3μM puromycin.

The pX458 plasmid was prepared to express Cas9 and a gRNA sequence (5′CCTGCCCTGAAGATGACCCT3’) targeting the region near the stop codon of the *MyD88* gene locus. Additionally, the donor pUC19 plasmid carrying a GFP gene flanked by approximately 800 bp homology arms was prepared. RAW264.7 cells were transfected with these two plasmids via electroporation and grown for two days. The first sorting was performed based on GFP signal using flow cytometry. After one week, a second round of sorting was conducted to isolate knock-in single clones. The clones with GFP knock in were validated by PCR and sequencing.

### 2.5 Live cell imaging

Myd88-GFP knock-in RAW264.7 cells were grown overnight in a 4-well glass dish. LPS (100 ng/mL), IL-1β (100 ng/mL), Pam2CSK4 (100 ng/mL), Pam3CSK4 (100 ng/mL), flagellin (100 ng/mL) were added to the dish before imaging under a Nikon TIRF microscope.

### 2.6 Protein expression and purification

The GST-MyD88 and MBP-MyD88 proteins were expressed and purified in BL21(DE3) *E.coli* strain. The bacteria were cultured in Luria-Bertani (LB) medium at 37 °C until the OD600 reached 0.6–0.8, and then the protein expression was induced by addition of 0.1 mM Isopropyl-β-d-thiogalactopyranoside (IPTG) at 16 °C overnight. The GST-MyD88(20–296) expressing bacteria were collected, lysed in lysis buffer (1x PBS, pH 7.4, supplemented with 1% Triton X-100 and protease inhibitor cocktail) using ultrasonication, then centrifuged at 13, 000 rpm for 60 min at 4 °C. The supernatant was filtered through 0.45-μm filters and loaded onto Sepharose 4B affinity gel. The protein was eluted with elution buffer (20 mM Tris, 150 mM NaCl, 20 mM glutathione, pH 8.0). After removing the GST tag with the PreScission protease, the proteins were further purified using Superdex 200 size exclusion chromatography. The fractions corresponding to 7–8 ml elution volumes were collected for TEM and cryo-EM observation. The MBP-MyD88 expressing bacteria were lysed in HEPES lysis buffer (25 mM HEPES, 500 mM NaCl, 20 mM imidazole, pH 7.5) supplemented with the protease inhibitor cocktail and 1 mM PMSF. The lysates were centrifuged at 13,000 rpm for 60 min at 4 °C. The supernatant was loaded onto a Ni sepharose 6 fast flow column. The proteins were washed with HEPES lysis buffer with imidazole gradient from 20 mM to 50 mM, then eluted with HEPES lysis buffer with 500 mM imidazole, and further purified using Superdex 200 size exclusion chromatography.

### 2.7 Negative-stain election microscopy

For TEM observation, the proteins were loaded onto a glow-discharged carbon-coated copper grid for 2 min, and then stained with 2% uranyl acetate for 15 s. The negatively stained samples were observed with a TEM (Hitachi H-7650B, Japan) operating at 80 kV with a magnification of 200,000×.

### 2.8 Cross-linking

HEK293T cells were transfected with FLAG-MyD88 plasmids and cultured for 20–24 h. The cells were washed twice with PBS, subsequently incubated with 2 mM DSS in PBS for 30 min, and then lysed with protein loading buffer and subjected to SDS-PAGE.

### 2.9 NF-κB luciferase activity assay

HEK293T were transfected with indicated plasmids (MyD88 plasmid, luciferase reporter plasmid) and cultured for 20–24 h, and then lysed with passive lysis buffer. The cell lysate was centrifuged for 10 min at 4 °C at 12,000 g. Luciferase reaction buffer (Promega, E1483) was added to the supernatant. Fluorescence values were recorded with a microplate reader (Thermo Varioskan Flash) at 360 nm. The data were normalized to the empty vector control.

### 2.10 Secreted embryonic alkaline phosphatase (SEAP) reporter assay

The HEK-Blue™ hTLR4 and HEK-Blue™ hTLR5 stable cells were cultured overnight and then stimulated with the indicated LPS or Flagellin for 18 h. 50 μL of the cell culture supernatant from each well were transferred to a new 96-well plate, and subsequently treated with 50 μL of QUANTI-Blue (InvivoGen) buffer. The plate was incubated at 37 °C in the dark for 30 mins, and then the absorbance at 620 nm was measured using a microplate reader (Thermo Scientific).

### 2.11 Turbidity assay

The MBP-tagged MyD88 proteins were diluted in 25 mM HEPES buffer at the indicated concentrations. After the addition of TEV protease, the turbidity was continuously detected at 350 nm for 90 min with an interval of 1 min at room temperature. The results were analyzed using Excel software.

### 2.12 RT-qPCR analysis

Cells were seeded in 12-well plates and treated as demonstrated in the figure legends. The total RNA was extracted using the TIANGEN RNAsimple Total RNA Kit. The RNA was reverse transcribed using the NovoScript Plus All-in-One 1st Strand cDNA Synthesis SuperMix, and the cDNA was analyzed by qPCR with NovoStart SYBR qPCR SuperMix Plus on a Bio-Rad T100 thermal cycler. Internal controls were GAPDH (human). The Primer sequences are listed in the supplementary materials.

### 2.13 FRET assay

293T/17 cells were transfected with the indicated plasmids and grown on a coverslip for 20–24 h. Cells were fixed with 4% paraformaldehyde and then mounted onto a glass slide. FRET efficiencies were calculated using the photo-bleaching FRET method. The fluorescence of the acceptor (MyD88-mCherry) was bleached to approximately 10% of the original fluorescence. The images of the donor (IRAK4-EGFP) before and after photo-bleaching were taken. The fluorescence of the donor before and after photo-bleaching was defined as I_DB_ and I_DA_ separately. The FRET efficiency (E) was calculated as follows:

(1) $$E=1-I_{\text{DB}}/I_{\text{DA}}$$

### 2.14 Computational simulation

Model preparation: The full-length MyD88 (UniProt: Q99836) was constructed in trRosetta [26], which generated four primary structures containing similar conformations in MyD88^DD^ and MyD88^TIR^ yet differing in orientation of MyD88^ID^. The full-length filament structure of MyD88 22-mer was built based on the MyD88DD filament (PDB: 6I3 N) with PyMOL (Schrödinger, LLC) and prepared in an aqueous system in which the MyD88 22-mer can repeat in the z-direction of a periodic box to form an extended filament. In systems for studying the assembly of MyD88 and mutated monomers, six MyD88 monomers were randomly placed in a dispersed state (Fig. S2). To compare the impact of mutations, systems containing only one monomer were also simulated (Fig. S2). We prepared all initial aqueous systems by VMD [27], in which MyD88 monomer(s) was/were solvated in ∼130,000 TIP3P water molecules, counter ions, and 0.10 M NaCl, totaling 127,000∼500,000 atoms in a periodic box of 110×110×110∼200×200×160 Å [28]. A summary of the simulation systems is provided in Table S1.

Simulation setup and trajectory analysis: All simulations were performed with the CHARMM36m-cmap force field [28]. Each system went through energy minimization, 25 ps dihedral restraint isothermal-isovolumetric (NVT) equilibration. Then, the system continued to relax in hundreds of nanosecond molecular dynamics (MD) simulations under isothermal-isobaric ensemble (NPT) using the AMBER18 package with GPU acceleration [29]. MD production runs were performed in the NPT ensemble (310K, 1 bar, Langevin dynamics thermostat and Monte Carlo barostat) with a time step of 2 fs. All lengths of bonds to hydrogen were constrained with SHAKE. The particle mesh Ewald (PME) technique was used for the electrostatic calculations. The van der Waals and short-range electrostatics were cut off at 12.0 Å with a switch at 10.0 Å. Trajectory analysis was performed using either “cpptraj” [30] or TCL scripts implemented in VMD [28], then further processed and plotted by Matplotlib [31]. Structural alignments were shown by PyMOL (Schrödinger, LLC).

### 2.15 Quantification and statistical analysis

Statistical analyses of the data were performed using GraphPad Prism 8 software. The one-way ANOVA with Tukey post hoc test was performed for multiple comparisons. *P* < 0.05 were considered to be of statistical significance.

## 3 Results

### 3.1 MyD88 assembles into filaments

**Fig. 1:**
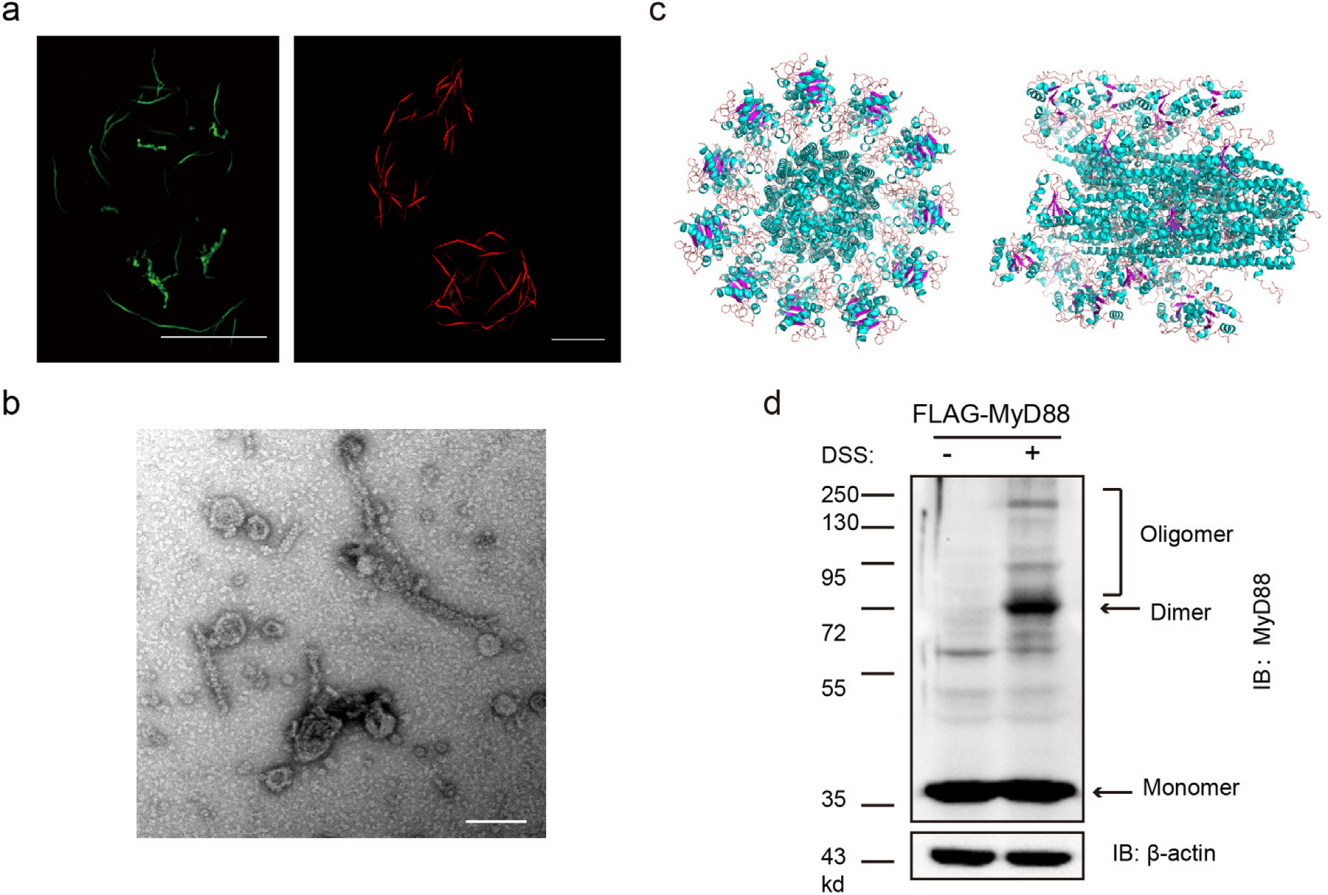
**MyD88 formed filament structures.** (a) Overexpressed MyD88-EGFP (left panel) or MyD88-mCherry (right panel) formed filament structures under a structured illumination microscope (SIM). (b) The MyD88 full-length protein was loaded onto a copper grid, stained with 2% uranyl acetate and observed under the transmission electron microscope. (c)The simulated MyD88 full-length assembly in top view projection (left) and in side view projection (right). (d) The FLAG-MyD88 plasmids were transfected into HEK293T cells. 24 h after transfection, the cells were treated with or without DSS for 30 min, then lysed with protein loading buffer and subjected to SDS-PAGE. The scale bars were 10 μm in (a), 100 nm in (b).

To address whether MyD88 can form filamentous structures, we first overexpressed full-length MyD88-EGFP and MyD88-mCherry in HeLa cells for observation at the cellular level (Fig. 1a). Both EGFP-tagged and mCherry-tagged MyD88 formed filamentous structures at micrometer length with approximately 100 nanometers in diameter. Of note, the filaments had varying lengths, diameters and branches. To rule out the possibility that MyD88 co-localized with the cytoskeleton, we stained β-Actin by phalloidine in MyD88-mCherry overexpressed cells with no co-localization observed (Fig. S1a). Similarly, MyD88-EGFP signals showed no obvious overlapping with α-tubulin (Fig. S1b). To validate the observation of MyD88 filament in immune cells, we also generated a MyD88-GFP knock-in RAW264.7 cell lines (Fig. S1c). However, we observed the formation of a droplet-like structure of MyD88 after TLR agonist stimulation in the cells (Fig. S1d).

To determine whether MyD88 alone formed filaments, we purified the recombinant GST-MyD88(20–296) protein, followed by removal of the GST tag. There are fiber-like structures several hundred nanometers long in the negatively stained protein samples under transmission electron microscopy (TEM) (Fig. 1b). Different lengths and diameters of MyD88 filaments were observed at the cellular level compared with the reconstituted system, likely due to different MyD88 concentrations.

To date, no full-length MyD88 structure has been deposited in the Protein Data Bank (PDB). Reported MyD88 structures were limited to the MyD88^DD^ in complex with the DDs of IRAK4 and the DDs of IRAK2 (PDB: 3MOP) or the MyD88^TIR^ alone (PDB: 2Z5 V, 4DOM, 4EO7, 2JS7, 7BER, 7L6 W). Recently, a cryo-EM structure of MyD88^DD+ID^ was reported to display a helical filament structure (PDB: 6I3 N) [32]. Based on this cryo-EM structure (PDB: 6I3 N), we constructed the MyD88 full-length filamentous structure and simulated the stability using microsecond molecular dynamics simulations. The results exhibited that the full-length MyD88 constituted a ring-like structure, with 11 monomers in every circle (Fig. 1c). The MyD88^DD^ and MyD88^ID^ formed the core components in the ring-like structure, with the MyD88^TIR^ surrounding the periphery. MyD88 further elongated into a helical filament by a package of eleven-mer ring-like structures in a way similar to the ‘railway carriage’. The assembly of the ring-like structure may serve as a platform for downstream kinase recruitment. Our data indicate that MyD88 filament assembles as a result of protein self-interaction and high-order self-oligomerization. To support this, we captured the MyD88 dimers and oligomers by performing the disuccinimidyl suberate (DSS) cross-linking experiment (Fig. 1d). Taken together, the cellular and reconstituted experiments data suggest MyD88 could self-assemble into dimers, oligomers, and supramolecular filaments.

### 3.2 Domains and key amino acids affecting MyD88 filament assembly

**Fig. 2:**
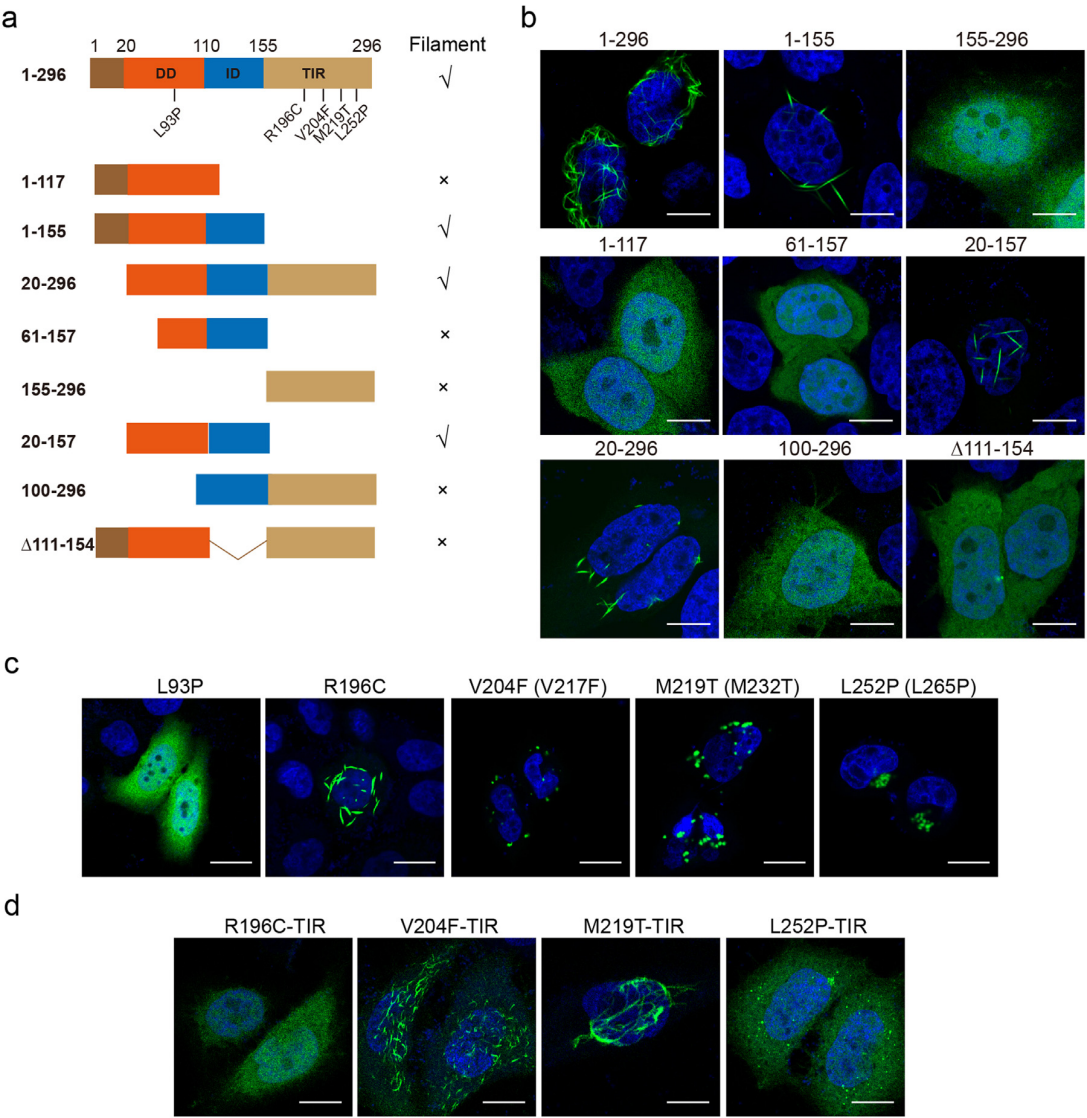
**Domains and sites important for the filament formation.** (a) Schematic diagram of MyD88 domains and mutations, and their abilities to form filaments. (b-d) MyD88-EGFP truncations, deletions (b), mutants (c) and TIR–domain-only mutants (d) were overexpressed in HeLa cells. After 20–24 h, the cells were fixed with 4% paraformaldehyde, stained with 4′,6-diamidino-2-phenylindole (DAPI), then sealed with mounting buffer, and observed under a confocal microscope. The scale bars in (b), (c) and (d) were 10 μm.

Next, we set out to identify the domains needed for the MyD88 filament formation. A series of MyD88-EGFP truncations and deletions were generated to dissect the domains necessary for filament formation (Fig. 2a). The overexpressed full-length MyD88 (1–296), MyD88 (1–155), MyD88 (20–157) or MyD88 (20–296) formed filamentous structures in HeLa cells (Fig. 2b). It was worth noting that MyD88 (1–155) and MyD88 (20–157) truncations without MyD88^TIR^ produced shorter filaments lacking branches compared to full-length MyD88. Shorter truncations such as MyD88 (1–117), MyD88 (61–157), MyD88 (100–296), and MyD88 (155–296) failed to form filaments but instead distributed in a diffused manner (Fig. 2b). MyD88 without DD (100–296) or ID (∆111–154) remained in a status of diffused distribution (Fig. 2b), indicating the MyD88^DD+ID^ (res. 20–157) is the indispensable unit for the filament formation. Furthermore, MyD88^TIR^ (155–296) alone didn’t produce filamentous structures but assisted in filament branching and elongating (Fig. 2b).

A number of human diseases were associated with MyD88 mutations. The L93P and R196C mutations were found in patients with immunodeficiency [15] The V204F (V217F), M219T (M232T) and L252P (L265P) mutations were frequently found in patients with lymphomas [17] Interestingly, different self-assembly patterns were observed when different disease-associated mutants of MyD88 were overexpressed (Fig. 2c). The L93P mutant exhibited a diffused distribution without any filament. Given the L93P substitution occurs in the DD, it perhaps perturbs MyD88^DD^-MyD88^DD^ interactions. The R196C mutant remained filamentous structures but with fewer branches. The V204F, M219T and L252P mutants all presented as speckles. The V204F, M219T and L252P mutations within the TIR domain were all considered as hyper-activated forms of MyD88. Therefore, the transformation of filaments to speckles perhaps enhances protein-protein interaction. To testify the speculation, we investigated the distribution patterns of the TIR-domain-only mutants (R196C-TIR, V204F-TIR, M219T-TIR and L252P-TIR) (Fig. 2d). The R196C-TIR had no noticeable change of diffused distribution compared to wild-type TIR. However, V204F-TIR and M219T-TIR mutants both appeared as filaments. In comparison, the L252P-TIR mutant produced speckles. These observations suggest that V204F, M219T and L252P mutants are able to enhance the MyD88^TIR^ -MyD88^TIR^ interactions, thus converting the MyD88^TIR^ assembly from dimers to oligomers.

### 3.3 Disease associated mutants had different abilities to form filaments

**Fig. 3:**
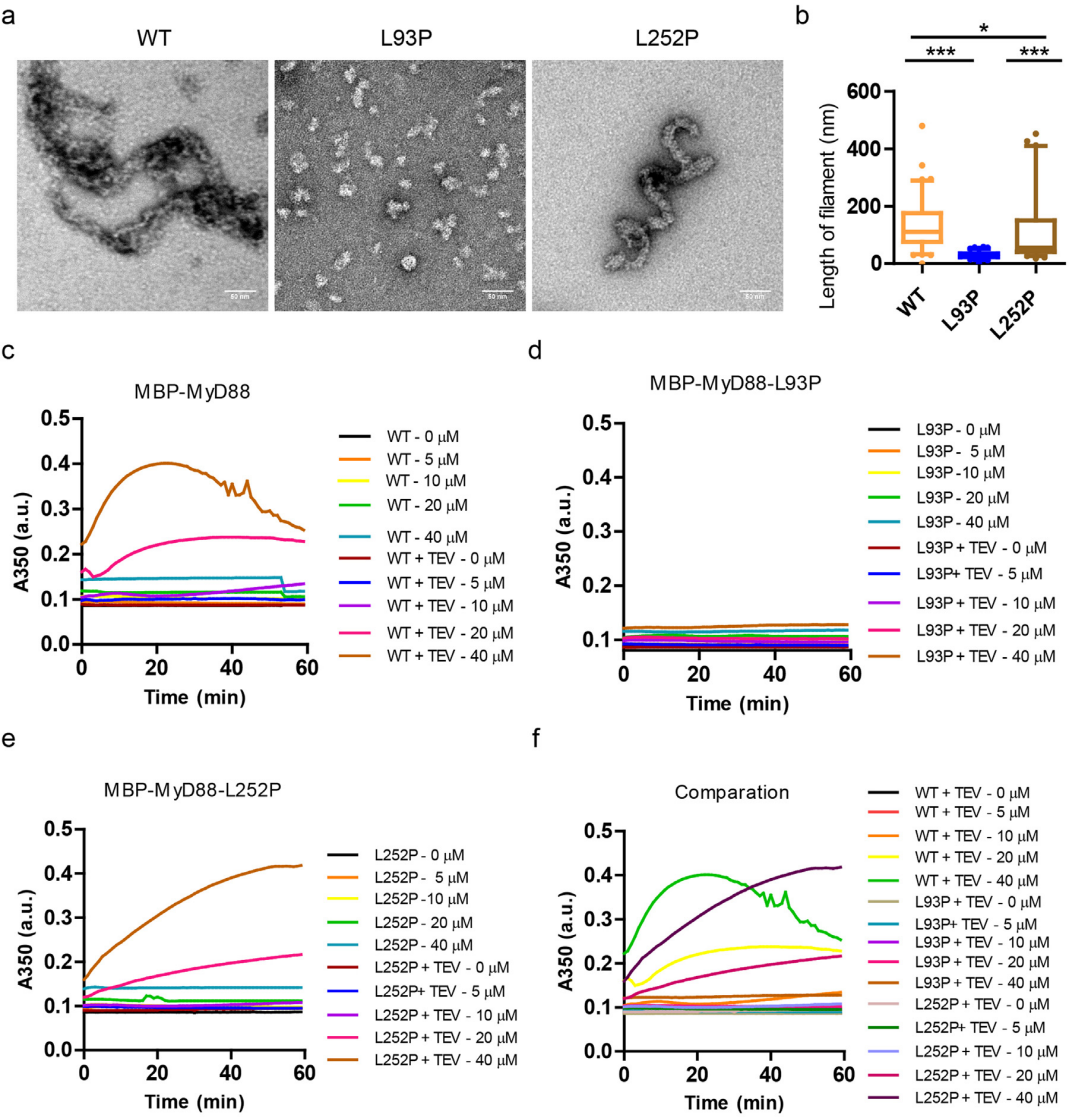
**Disease-associated mutants had different abilities to form filaments.** (a) MBP-tagged MyD88 wild type (WT), L93P and L252P proteins were loaded onto copper grids, stained with 2% uranyl acetate, and observed under a transmission electron microscope. Scale bar, 50 nm; (b) Statistical analysis of filaments formed by MBP-WT, MBP-L93P and MBP-L252P proteins. (c-e) MBP-WT (c), MBP-L93P (d) and MBP-L252P (e) proteins were diluted to indicated concentrations in 25 mM HEPES buffer, pH7.5, in the presence or absence of the TEV protease, and detected at 350 nm for absorbance. (f) Comparison of turbidity of MBP-WT, MBP-L93P and MBP-L252P proteins in the presence of TEV protease.

Functionally, the L93P mutant and the L252P mutant had opposite effects in the activation of NF-κB signal [15,17]. These critical amino acids might serve as a molecular switch in the TLR signaling pathway. To figure out the deeper connection between the different self-assembly morphologies of the MyD88 mutants and their responses to stimulations, we carried out experiments in the reconstituted system. MBP-tagged wild-type (WT), L93P and L252P MyD88 recombinant proteins showed different morphologies under the TEM. The MBP-MyD88-WT showed as twined bundles of several filaments; the MBP-MyD88-L93P displayed as spherical-like structures of several nanometers; the MBP-MyD88-L252P formed helical-like filaments (Fig. 3a). Statistical analysis revealed that the average length of filaments was shortened in a descending order from WT, L252P to L93P (Fig. 3b). This result uncovered that the L252P mutation impaired the ability to form long filaments, and the L93P mutation abolished filament formation. We further observed that removal of MBP-tag by the tobacco etch virus (TEV) protease turned the protein solutions turbid. The turbidity of WT, L93P and L252P solutions was measured at various protein concentrations in the presence or absence of TEV protease to evaluate their abilities of further growth (Fig. 3c–e). In the presence of TEV protease, WT and L252P solutions became turbid at a concentration of 20 μM. Meanwhile, the turbidity of L93P solution remained unchanged even at a high concentration of 40 μM. In comparison, the WT solution became turbid faster than L252P solution, while turbidity signals of MyD88 WT solution decreased over time. The turbidity of L252P solution showed a continuing growth trend after adding TEV protease 60 mins later (Fig. 3f). These data suggested that WT assembled rapidly; by comparison, L252P assembled steadily at a slower speed in response to PAMP or DAMP signals. A recent work using single-molecule fluorescence fluctuation spectroscopy reported that WT MyD88 can polymerize while L252P mutant forms oligomers at a very low concentration [33]. Our investigation of filament formation in vitro further demonstrates that WT assembles faster than L252P, but L252P assembles into a more stable form.

### 3.4 Disease associated mutations altered the interactions with downstream protein

**Fig. 4:**
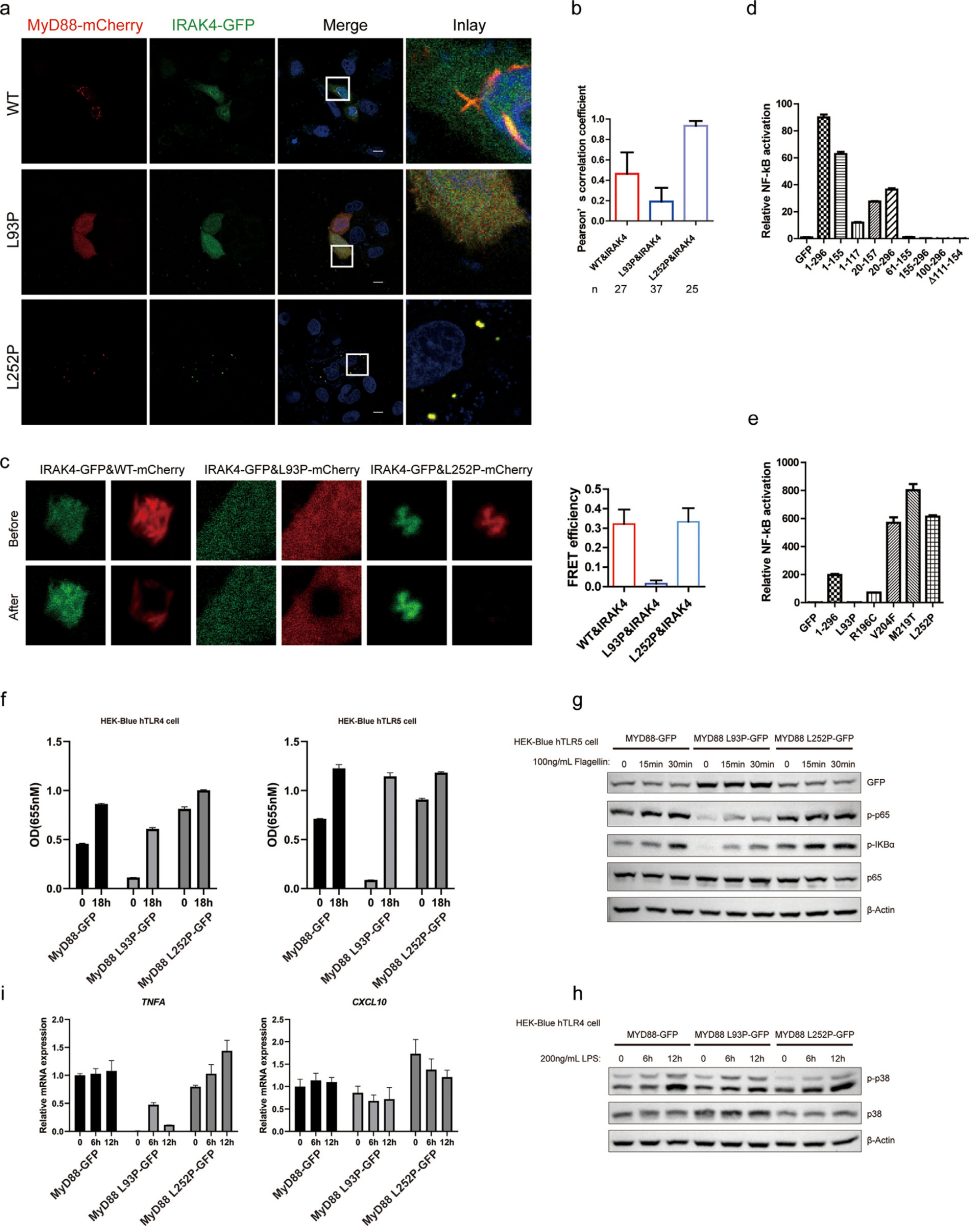
**MyD88 assembly mediated NF-κB signaling.** (a) The images of MyD88-WT-mCherry, MyD88-L93P-mCherry and MyD-L252P-mCherry co-expressed with IRAK4-EGFP. (b) Statistical analysis of Pearson’s correlation coefficient of proteins in (a). (c) The MyD88-WT-mCherry, MyD88-L93P-mCherry and MyD-L252P-mCherry plasmids were co-expressed with IRAK4-EGFP plasmids separately in 293T/17 cells. 24 h after transfection, the cells were fixed and mounted. Under Nikon A1 MP confocal microscope, the images of the acceptor (MyD88) and donor (IRAK4) were recorded before and after photo-bleaching. The FRET efficiencies were analyzed using ImageJ and Excel software. (d, e) Luciferase activity results of MyD88 truncations, deletions (d) and mutants (e). The corresponding proteins were overexpressed in 293T/17 cells. After 20–24 h, the cells were lysed with passive lysis buffer, reacted with luciferase reaction buffer, and recorded with a microplate reader. (f) SEAP assay of supernatants from the indicated stable cell lines, collected 18 h post-treatment, measured at 655 nm. MyD88-GFP, MyD88 L93P-GFP, and MyD88 L252P-GFP were stably expressed in HEK-Blue™ hTRL4 cell and HEK-Blue™ hTRL5 cells. (g) Immunoblot analysis of the indicated proteins in hTLR5 stable cell lines treated with 100 ng/mL Flagellin for the indicated durations. (h) Immunoblot analysis of the indicated proteins in hTLR4 stable cell lines treated with 200 ng/mL of LPS for the indicated durations. (i) RT-qPCR analysis of TNFA and CXCL10 mRNA expression in hTLR5 stable cell lines treated with 100 ng/mL Flagellin for the indicated durations. Data are mean ± SEM. Unpaired *t*-test; n.s., not significant; **, *P* < 0.01; ***, *P* < 0.001; ****, *P* < 0.0001.

To explore how the disease-associated mutations interfered with the downstream signals, we conducted co-expression and Förster resonance energy transfer (FRET) experiments. Interestingly, IRAK4 alone distributed in a diffused manner but partially co-localized with filaments formed by MyD88 (Fig. 4a). IRAK4 had reduced co-localization with L93P signals. By contrast, IRAK4 almost completely co-localized with L252P (Fig. 4a). Pearson’s correlation coefficient analysis further validated this co-localization tendency (Fig. 4b). Furthermore, the L252P mutant exhibited the strongest interaction with IRAK4 compared with WT and L93P (Fig. 4c). These results suggest that the L93P mutation blocks the interaction with IRAK4, which instead is strengthened by the L252P mutation. Meantime, no co-localization of overexpressed IRAK1 and MyD88 mutants was observed. It is likely that IRAK1 indirectly interacts with MyD88 or certain stimulations are required for their interaction.

Next, we performed NF-κB reporter luciferase activity assays to test the abilities of different MyD88 truncations, deletions and mutations to transduce NF-κB signals. Compared with full-length MyD88 (1–296), MyD88 (1–155) truncation showed less but still considerable activation of NF-κB signaling, and the MyD88^DD^-only (1–117) construct had significantly lower activation of NF-κB signaling (Fig. 4d). The MyD88^TIR^ (155–296) failed to activate the NF-κB signal. Deletion of MyD88^ID^ (∆111–154) also blocked the activation (Fig. 4d). These data suggest that the MyD88^DD+ID^ is the minimum unit to activate NF-κB downstream signals, while the MyD88^TIR^ also contributes to the signal activation. Remarkably, the L252P, V204F, M219T mutants significantly augmented the NF-κB signal, which was impaired by the R196C mutant, and abrogated by the L93P mutant (Fig. 4e). Intriguingly, despite forming filaments, the R196C mutant exhibited only a partial immune response. We investigated the interaction between R196C and IRAK4. The FRET results revealed a lower FRET efficiency of approximately 20% (Fig. S1c), suggesting that this mutation causes subtle structural changes that weaken its interaction with IRAK4.

Next, we explored the downstream signaling of disease-related mutations. In SEAP assays using HEK-Blue™ hTLR4 cells, we found that L252P mutant induced stronger activation both in the presence and absence of LPS (Fig. 4f). In contrast, the L93P mutant showed almost no activation without flagellin stimulation, but produced a comparable SEAP signal upon flagellin stimulation (Fig. 4f). These results may be explained by differences in their affinity for upstream TLR4 and TLR5 under various physiological conditions. The phosphorylation levels of downstream proteins IκBα and p65 mirrored the of NF-κB activation levels observed in WT, L93P and L252P mutants (Fig. 4g). Similarly, the activation of p38 pathway by WT, L93P and L252P exhibited a similar trend (Fig. 4h). Finally, the differential activation of these pathways led to varying levels of cytokines production (Fig. 4i).

These data imply that mutations in DD directly interrupt downstream signaling, while mutations in TIR domain regulate the activation levels of downstream NF-κB, AP-1 and p38. To summarize, our data reveal that different MyD88 mutants have varied effects in activating NF-κB, AP-1 and p38, which may be relevant with their assembly behaviors and interactions with IRAK4.

### 3.5 Structural features of disease-associated mutants and assemblies

**Fig. 5:**
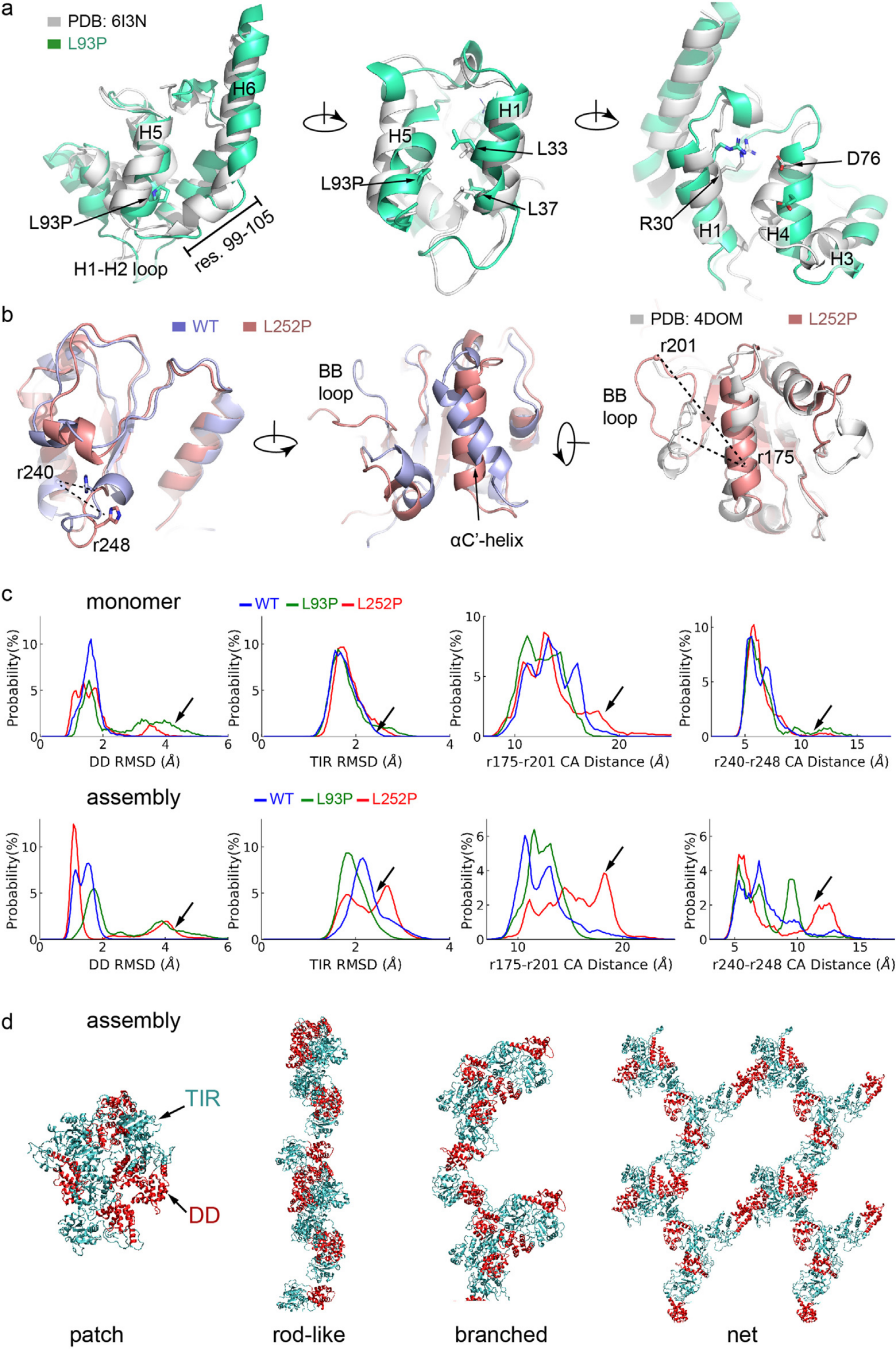
**Structural features of WT, L93P, and L252P full-length MyD88 in monomer and self-assembled oligomers.** (a) Structural superposition of L93P DD on the cryo-EM structure of MyD88^DD^ (PDB: 6I3 N). Unfolding of residues 99–105 and rearrangements between helices H1-H6 were observed. (b) Superposition of L252P TIR on cryo-EM structure of MyD88^TIR^ (PDB: 4DOM) and WT, respectively. The BB-loop and residues 246–252 in L252P became more extended than WT.(c) Upper and lower panels displayed statistical distributions in simulations of WT (blue), L93P (green), and L252P (red) monomer and self-assembly respectively for: backbone RMSD of DD superposed on 6I3 N; backbone RMSD of TIR superposed on 4DOM; BB-loop distance to its neighboring helix (measured by Cα distance between residues 175 and 201), H248 distance to its neighboring αC´-helix (measured by Cα distance between residues 240 and 248). (d) WT, L93P, and L252P assembled into patch, rod-like, branched, and net structures with DDs (res. 19–121) in red and the rest in cyan.

As the assembly differences between various MyD88 mutants were closely associated with their physiological activities, we further probed into the structural features and molecular mechanisms behind these distinct assembly behaviors. We carried out molecular dynamics simulations to examine the relationship between various structural features and self-assemblies of full-length WT, L93P, and L252P MyD88. In the DD, L93P generated a kink at the helix 5 (H5). Since the side chain of a proline residue is substantially smaller than that of a leucine residue, the mutation disrupted the original hydrophobic packing, resulting in the rearrangement of helices H1-H6 (Fig. 5a). As a result, the neighboring residues 99–105 of H6 tended to unfold in L93P mutant (Fig. S3a). The residues R30 and D76 in the WT, which were close to each other and tended to form hydrogen bond, stayed farther away in L93P mutant due to the rearrangement between H1 and H4. The conformational changes were also measured by the backbone root-mean-square deviation (RMSD) by aligning WT, L93P, and L252P MyD88 on the cryo-EM structure of MyD88^DD^ (Fig. 5c). L93P displayed a large population of RMSD over 3 Å (green line in Fig. 5c), while WT maintained a pretty stable DD structure with an RMSD of 0.9∼1.5 Å. The significant conformational change in the DD may impair the formation of filamentous structure. To summarize, L93P induced MyD88^DD^ conformational changes by altering the hydrophobic core and changing the relative distances of H1-H5, H1-H2 and H1-H4.

In the TIR domain, L252P changed its connecting helical turn at residues 246–252 into a random coil with decreased helicity (Fig. S3a), compared with WT (PDB: 4DOM and 4EO7). The residue H248 in L252P mutant stayed further away from the neighboring αC´-helix (measured by Cα distance between res. 240 and 248, Fig. 5c), making it more accessible in the environment. The unfolding in the region of residues 246–252 was also observed as a populated conformation in L252P mutant by a previous study focusing on the TIR domain of MyD88 [13]. The overall conformational change of TIR was more significant in the L252P mutant, particularly in the assembly simulations as indicated by the RMSD of TIR (Fig. 5c). The conformational change in the αC´-helix and the helical turns that connect to the BB-loop (Fig. 5b) could accelerate the dissociation of the BB-loop indicated by increased Cα distance between residues 175 and 201 (Fig. 5b,c). Therefore, the BB-loop in L252P mutant became more flexible and more exposed to the environment. Due to the important role of BB-loop in MyD88^TIR^ -MyD88^TIR^ dimer packing, the extended BB-loop on L252P mutant may enhance the MyD88^TIR^ -MyD88^TIR^ interaction and conformation transition, making it more favorable for downstream protein recruitment and activation.

The disease-associated mutations not only brought in local structural changes, but also affected the morphology and structure in self-assembly. Upon assembling of WT, L93P, and L252P MyD88, we observed four different assembly structures: patch-like, rod-like, branch-like, and net-like (Figs. 5d and S4a-c). However, these mutants displayed distinct structure preferences. For example, WT tended to aggregate into elongated branched oligomers (Fig. S4a); L93P mutant contained more rod-like oligomers (Fig. S4b, d); L252P mutant was populated in patch-like oligomers (Fig. S4c, d). The data mutually echoed our previous results at the cellular level and in the reconstituted system. Different assembly preferences may be attributed to the inter-protein interactions altered in different mutants. The L93P mutant that formed rod-like assemblies was populated in inter-protein MyD88^DD^-MyD88^DD^ contacts. The L252P mutant that formed more patch-like oligomers exhibited more inter- protein MyD88^TIR^ - MyD88^TIR^ contacts, while WT with more branched oligomers displayed more inter- protein MyD88^DD^ -MyD88^TIR^ contacts (Fig. S3b). These results indicate that the filament branching may depend on MyD88^DD^-MyD88^TIR^ interactions more than MyD88^DD^-MyD88^DD^ or MyD88^TIR^-MyD88^TIR^ interactions. Our computational simulation studies revealed a new type of MyD88 interaction during filament formation, i.e., the MyD88^DD^-MyD88^TIR^ interaction. As it was reported that the MyD88^DD^-MyD88^TIR^ interaction could auto suppress MyD88 activity [34], the reduced MyD88^DD^-MyD88^TIR^ contacts in L252P mutant coincided with its enhanced activity. With increased MyD88^TIR^-MyD88^TIR^ contacts, L252P mutant displayed more exposure of surface area at the H1-H2 loop and at the TIR domain (Fig. 5c). Interestingly, the H1-H2 loop has a major impact on MyD88 interaction with IRAK4 [4]. Thus, the result revealed a possible mechanism of the strengthened interaction between L252P and IRAK4.

Overall, these simulations were consistent with the TEM results. Moreover, these molecular insights deepened our understanding of the structural changes resulted from the L93P and L252P mutations. In morphology, deformation of MyD88^DD^ by L93P mutation disrupts a higher degree assembly of MyD88. By comparison, L252P mutant may adopt stronger MyD88^TIR^ -MyD88^TIR^ interactions, hence prefers the formation of shorter assemblies with more exposed DDs, and in turn, strengthens downstream NF-κB signaling.

## 4 Discussion

The adaptors play important roles in innate and adaptive immunity [35,36]. They respond in a flexible and dynamic manner to environmental challenges [37]. Our results suggest that MyD88 possesses the ability to self-assemble into filaments. The MyD88^DD+ID^ is the minimum unit for the MyD88 filament extension. The MyD88^TIR^ functions in synergy with MyD88^DD+ID^ to promote filament branching and elongating. The oligomerization of MyD88 into filaments probably acts as a critical platform to recruit sufficient downstream molecules, thus amplifying the immune signals and finally initiating anti-pathogen responses in a short time. Likewise, several adaptor proteins in immune signaling adopt a common mode of oligomerization and assembly to defend against invading bacteria or viruses, including ASC (apoptosis-associated speck-like protein containing a CARD) [38], MAVS (the mitochondria anti-viral signaling protein) [39], and the RIP1/RIP3 complex [40]. Common to these adaptor proteins, despite having different structure domains, they all formed filaments in signal transduction. The reported structures of three adaptor proteins in TLR signaling—TRIF, TIRAP and TICAM—have all been shown to adopt filamentous structures. This type of assembly may represent a common mechanism among certain kinds of adaptor proteins. Recently, phase separation has emerged as another paradigm in signal transmission. For example, the TIFA-TRAF6 complex was found to condensate into droplet-like structures during ADP-hep stimulation [41]. Similarly, TRAF6-NEMO also formed phase condensates in response to PAMPs or DAMPs [42]. In adaptive immunity, the adaptor protein LAT recruits and interacts with multiple proteins through phase separation during T cell activation. MyD88 recently was reported to produce speckle-like structures upon activation, a phenomenon similar to what we observed in our knock-in cells [43], likely due to extensive interactions with associated proteins in cells. Additionally, STING, an adaptor protein crucial for DNA sensing in innate immunity, modulates the signaling pathway from cGAS to downstream TBK1 [44]. STING has been shown to form oligomers at molecular level [45] and phase separator at the organelle level [46]. The different states and functions of adaptor proteins warrant further investigation from structural, cellular and dynamic perspectives. Whether the adaptors form rigid filaments or dynamic phase separators remains a question to be explored.

Since MyD88 plays a vital role in innate immunity, the impairment and the elevation of MyD88 activities could perturb the physiological functions of cells. We find that the L93P mutant loses the ability to form filaments, disables interactions with IRAK4, and thus fails to activate NF-κB signals. Conversely, L252P mutant assembles into speckle-like structures with stronger interaction with IRAK4, resulting in enhancement of NF-κB signal activation. At the molecular level, our results reveal that the L93P mutation disrupts an α-helix in H6, contributing to the rearrangement of MyD88^DD^ hydrophobic core; the L252P mutation also unwinds a short helix between residues 246–252, leading to more surface exposure of the BB-loop. The L93P mutant has increased MyD88^DD^-MyD88^DD^ interactions while less MyD88^TIR^-MyD88^TIR^ interactions. This allosteric effect was also observed at other mutation sites in MyD88, such as the S34Y and R98C mutations [47], indicating a similar mechanism of MyD88 related non-synonymous SNP diseases. The L252P (L265P) mutant is reported to have a stronger interaction with IRAK4 [17,48], likely because its modified conformation enhances MyD88^TIR^-MyD88^TIR^ interactions but weakens MyD88^DD^-MyD88^DD^ interactions, which increases its binding affinity towards IRAK4.

**Fig. 6:**
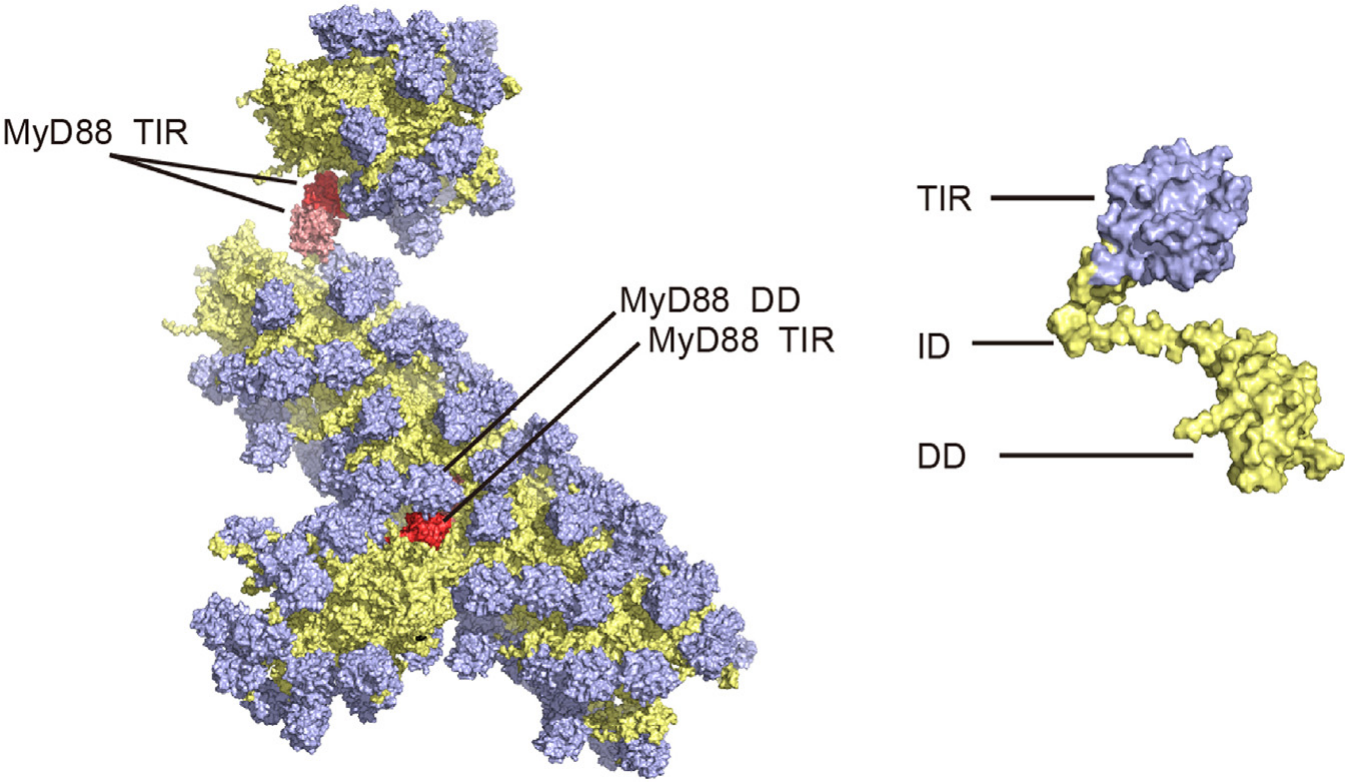
**Molecular model of the MyD88 filament assembly.** Schematic diagram of MyD88 filament assembly. The MyD88^DD+ID^ is the minimal unit for MyD88 filament assembly. MyD88^DD^-MyD88^DD^, MyD88^TIR^-MyD88^TIR^ and MyD88^DD^-MyD88^TIR^ interactions mediate the elongation and branching of filaments.

In this study, we proposed a model that MyD88 assembles into a supramolecular complex to amplify NF-κB signaling (Fig. 6). In this model, MyD88 protein first assembles into an 11-mer ring-like structure as the building block for further growth into filaments. Eleven MyD88^DD+ID^ molecules comprise the core of one filament unit with MyD88^TIR^ in the periphery region. The stacking of rings through MyD88^DD^-MyD88^DD^ interactions ultimately forms filaments. Moreover, through MyD88^TIR^-MyD88^TIR^ or MyD88^DD^-MyD88^TIR^ contacts, the filament can branch into a network. A recent study reveals that MyD88 oligomers require at least four MyD88 molecules to initiate sequential recruitment of IRAK4 and IRAK1 [49]. Furthermore, the depletion of IRAK4 in cells enlarges MyD88 oligomer sizes [49]. Combined with our findings, it suggests that MyD88 forms large filaments in a resting state and disperses into myddosome after recruiting IRAK4 and IRAK1. The droplets observed in RAW264.7 cell lines (Fig. S1d) are similar to the findings of a recent study [43], suggesting that the behavior of MyD88 may be influenced by the specific context of the stimulation and cellular environment. Further investigation is needed to elucidate the precise nature of these structures and their role in MyD88-mediated signaling pathways. Additionally, exploring the underlying mechanisms that differentiate filament and droplet formation could provide valuable insights into the dynamic behavior of MyD88 and its impact on immune cell function.

Mutations associated with MyD88 can lead to immunodeficiency or hyper-reactive immune responses. Over-activation of immune responses often releases excessive cytokines and causes autoimmune diseases or acute injuries to organs. Several inhibitors targeting MyD88^TIR^-MyD88^TIR^ dimers have been developed, including ST2825, T6167923, TJ-M2010–2, TJ-M2010–5 and LM9 [[50], [51], [52], [53], [54]]. These inhibitors are validated to effectively interrupt the formation of MyD88 dimers, thus alleviating inflammatory responses. MyD88 dimerization inhibitors are administered in treatment with reperfusion-induced progressive renal injury model [52], traumatic brain injury in mice, and Staphylococcal enterotoxins induced toxic shock in mice [54,55]. Recently, a transgenic mouse model bearing intrinsic MyD88 L252P mutation and additional BCL2 copies has been constructed and evaluated [56]. Knittel et al. [47] reported that B-cell-specific conditional expression of Myd88 L252P leads to the development of diffuse large B-cell lymphoma in mice. Another mouse model with continuous L252P expression in CD19-positive B-cells exhibited phenotypes resembling Waldenström macroglobulinemia syndrome (WM) [57]. These in vivo studies confirmed L252P as a driver mutation in the development of DLBL and WM. Our finding provides a new perspective on the molecular mechanisms underlying L252P-driven malignant diseases by examining protein assembly and structural disturbances. Developing drugs that target the self-interaction of L252P or the L252P-IRAK4 interaction could offer a novel and promising therapeutic strategy for treating DLBCL and WM. Given the close association between MyD88 dysfunction and various human diseases, investigating its structure and signaling mechanism holds promising potential for the development of future therapeutics. A growing number of inhibitors targeting the interaction between MyD88 and TLRs have been reported [[58], [59], [60]]. However, there are currently no reported co-crystallization structures of MyD88 with its inhibitors, apart from those based on artificial intelligence models [61]. Structural insights into MyD88 could significantly aid the development of specific drugs targeting its self-assembly or the MyD88-IRAK4 interaction, potentially offering therapeutics for a wide range of diseases.

## Credit authorship contribution statement

H.Y. and J.W. conceptualized the study. J.W., X.Z. and C. L. performed most of the experiments. Y. C., S.S. and R. Z. performed supporting experiments. G.L. and H.Y. supported and supervised the study. J.W., X.Z., C. L., Y. C., S.S., R. Z., G.L. and H.Y. wrote and revised the manuscript.

## Declaration of competing interest

The authors declare that they have no conflicts of interest in this work.

## Appendix Supplementary materials

Image, application 1
