# Supplementary material for: MyD88 self-assembles into supramolecular filaments to amplify NF-κB signaling
Source: Fundam Res. 2025 Feb 25;6(4):2319–30. doi: 10.1016/j.fmre.2025.02.010 (PMC13424689; doi:10.1016/j.fmre.2025.02.010)
Supplement: Supplementary file 1 [file mmc1.docx]

Supplementary Materials for

MyD88 self-assembles into supramolecular filaments to amplify NF-κB signaling

Jia Wang^a,1^, Xincheng Zhong^a,1^, Chenyi Liao^b,1^, Yuchen Zhang^a^, Siqi Shen^a^, Ran Zhang^c^, Guohui Li^b,^*, and Hang Yin^a,^*

*Corresponding author: Guohui Li (ghli@dicp.ac.cn) or

Hang Yin (yin_hang@tsinghua.edu.cn)

**The PDF file includes:**

Fig S1. Characterization of MyD88 Localization in HeLa and RAW264.7 cells.

Fig S2. Initial setup for monomer and assembly simulations.

Fig S3. Simulations of self-assembly MyD88 WT (blue), L93P (green), and L252P (red).

Fig S4. Oligomers in simulations of self-assembly.

Table S1. A summary of MD simulations.

Table S2. Primers for qPCR.

Table S3. Key resources table.


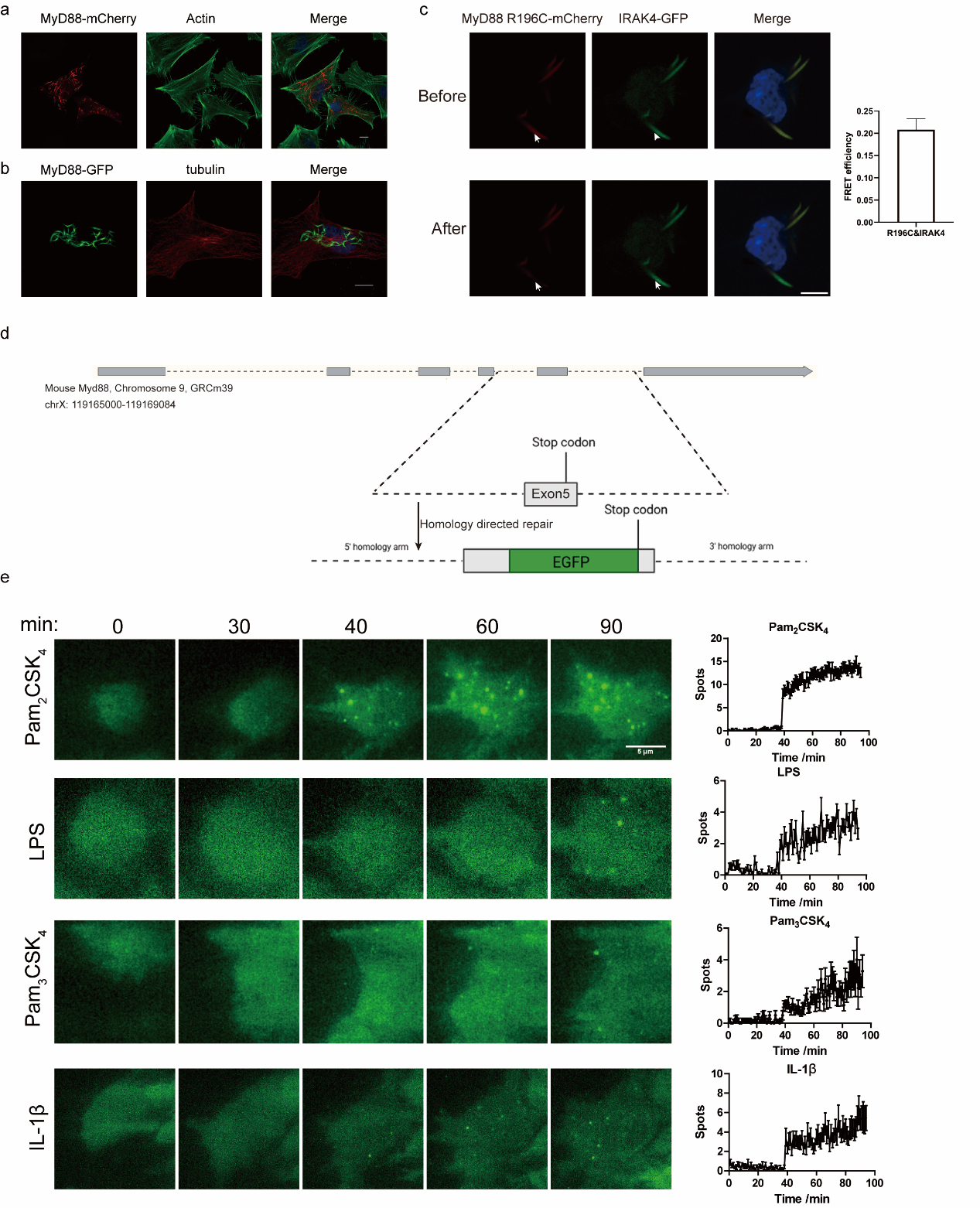
 Figure S1. Characterization of MyD88 Localization in HeLa and RAW264.7 cells. (a) Images of MYD88-mCherry and actin filament stained with phalloidine in HeLa cells. (b) Images of MyD88-EGFP and tubulin filament stained with tubulin antibody in HeLa cells. Scale bars, 10 μm. (c) The MyD88 R196C-mCherry plasmid was co-expressed with IRAK4-EGFP plasmids in HEK293T cells. 24 h after transfection, the cells were fixed and stained with DAPI. Under Nikon A1 MP confocal microscope, the images of the acceptor (MyD88) and donor (IRAK4) were recorded before and after photo-bleaching. The FRET efficiencies were analysis using ImageJ and Excel software. Scale bars, 5 μm. (d) Diagram of generating Myd88-GFP knock-in RAW 264.7 cell line. (e) Live cell imaging of the Myd88-GFP RAW cells with indicated stimulation.


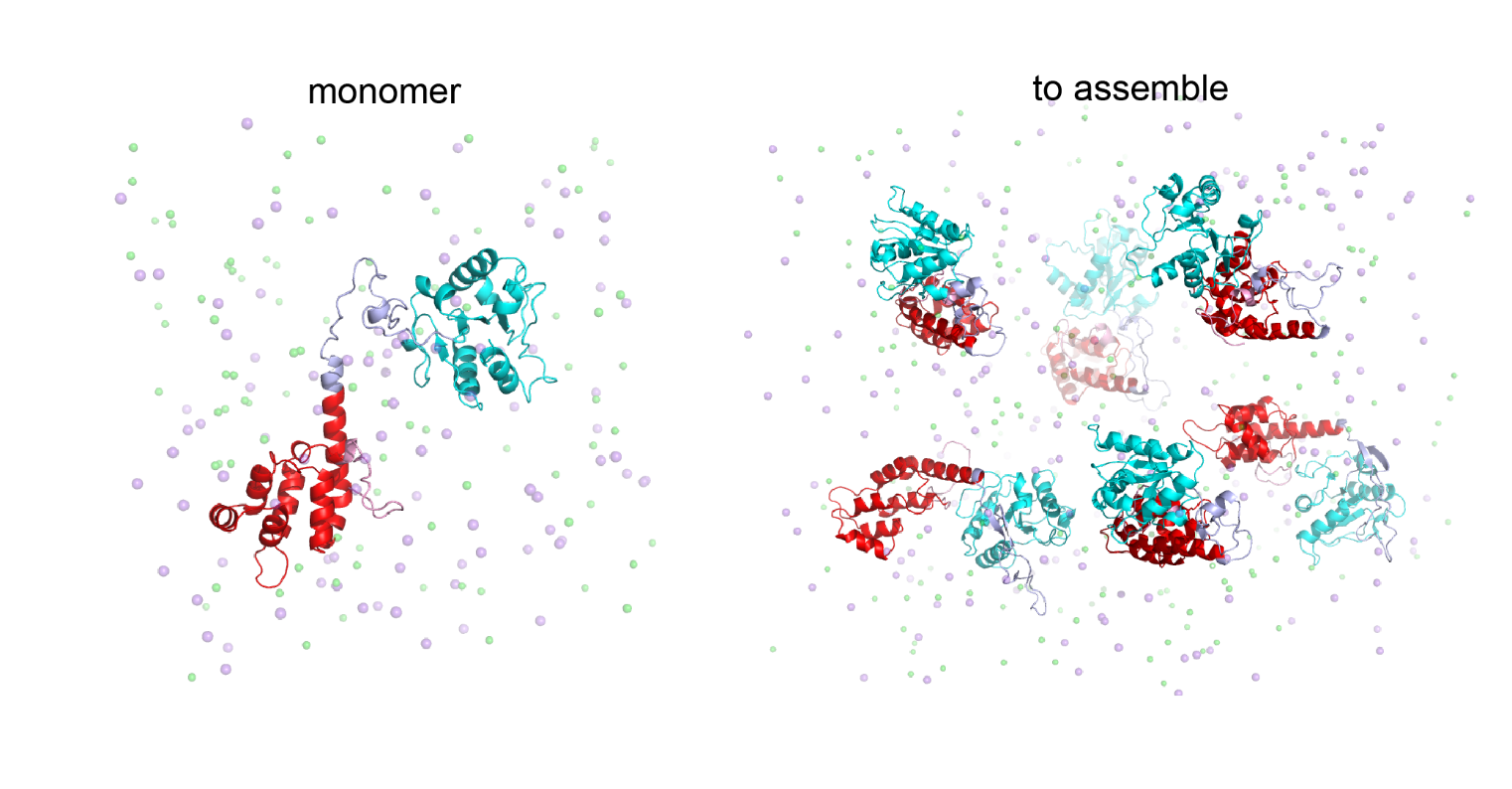


Figure S2. Initial setup for monomer and assembly simulations. DDs (res. 19-121) are in red and TIRs (res. 162-296) are in cyan; ions are displayed in sphere; water are not shown.


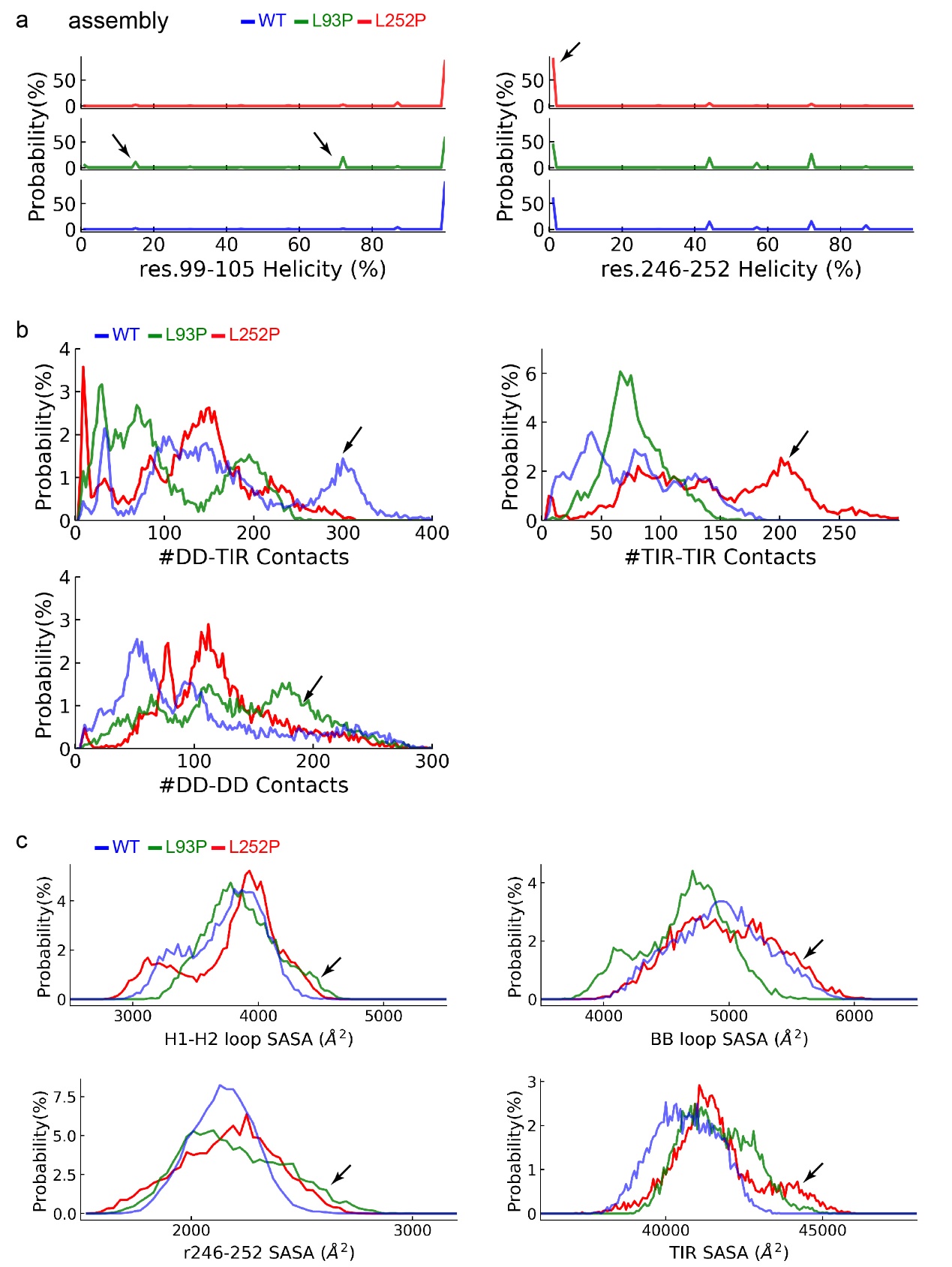


Figure S3. Simulations of self-assembly MyD88 WT (blue), L93P (green), and L252P (red) (a) Helicity percentages of res. 99-105 and res 246-252 in oligomers of WT, L93P, and L252P, respectively. Res. 246-252 in L252P mutant which tend to unfold (indicated by arrows), thus become more extended. (b) Statistical distribution of inter-protein contacts for the DD (res. 1-121) and TIR (res. 157-296), TIR and TIR, DD and DD in oligomers of WT, L93P, and L252P. (c) Statistical distributions of solvent accessible surface area (SASA) in H1-H2 loop (res. 39-46), BB-loop (res. 194-208), res. 246-252, and TIR (res. 162-296). Larger SASA indicates more exposure as indicated by arrows.


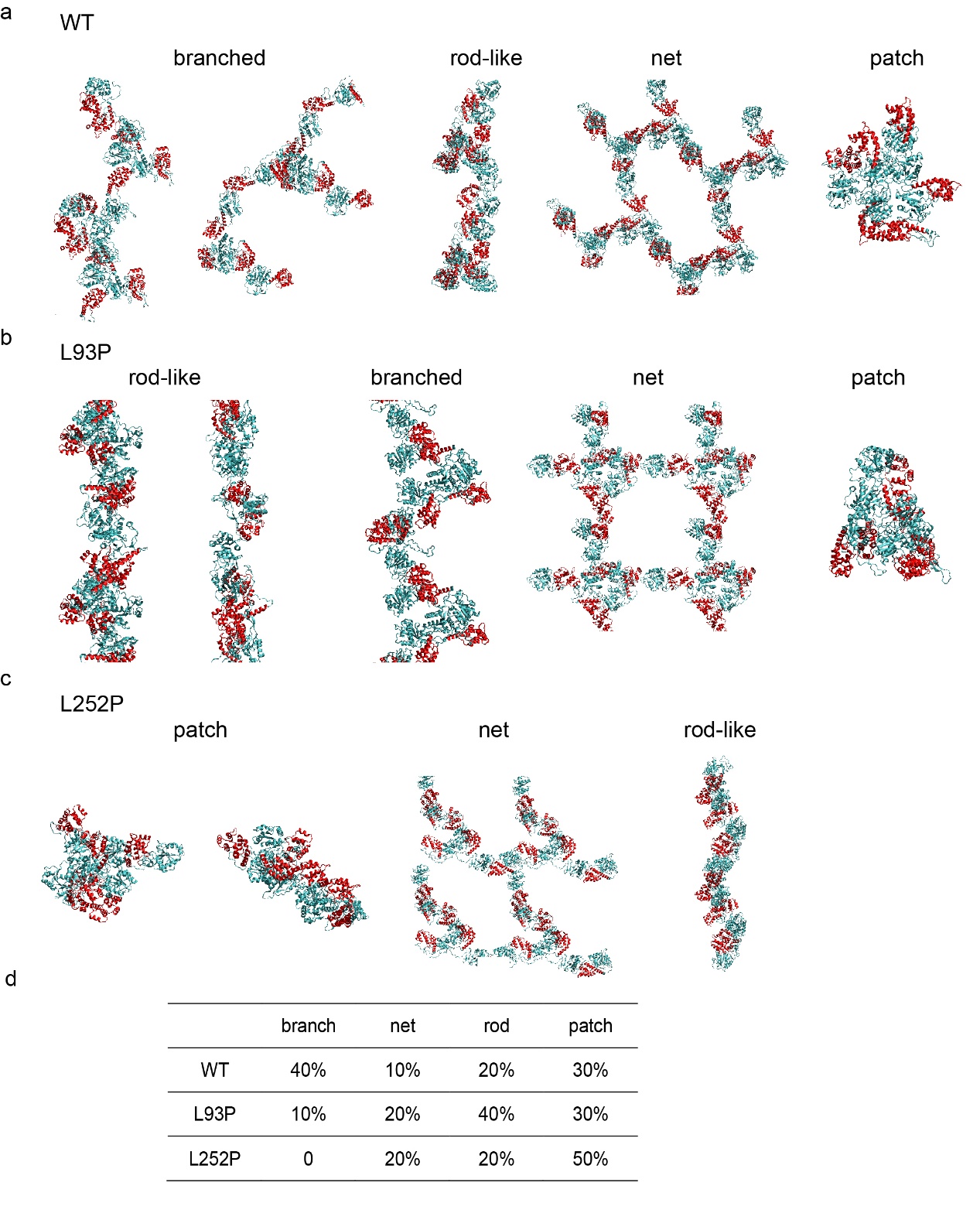


Figure S4. Oligomers in simulations of self-assembly (a) WT, (b) L93P, and (c) L252P MyD88 as patch, rod-like, branched, and net structures. DDs (res. 19-121) are in red and the rest are in cyan. (d) Percentage of different oligomer structures.

Table S1. A summary of MD simulations.

| Sys. | Num. of atoms | Box dimension (nm^3^) | Simulation length (ns) |
| --- | --- | --- | --- |
| WT 22-mer | 492775 | 200×200×131 | 1000×2 replicas |
| Mutated 22-mer | 492483 | 200×200×131 | 1000×2 replicas |
| WT monomer | 126678 | 110×110×110 | 800×10 replicas |
| L93P monomer | 126664 | 110×110×110 | 800×10 replicas |
| L252P monomer | 126664 | 110×110×110 | 800×10 replicas |
| six WT monomers | 386659 | 160×160×160 | 1200×10 replicas |
| six L93P monomers | 387345 | 160×160×160 | 1200×10 replicas |
| six L252P monomers | 387432 | 160×160×160 | 1200×10 replicas |

**Table S2. Primers for qPCR**

| GAPDH-F | ATGACATCAAGAAGGTGGTG |
| --- | --- |
| GAPDH-R | CATACCAGGAAATGAGCTTG |
| TNFA-F | ACTTTGGAGTGATCGGCC |
| TNFA-R | GCTTGAGGGTTTGCTACAAC |
| CXCL10-F | CCTTATCTTTCTGACTCTAAGTGGC |
| CXCL10-R | ACGTGGACAAAATTGGCTTG |

Table S3. Key resources table.

| REAGENT or RESOURCE | SOURCE | IDENTIFIER |
| --- | --- | --- |
| Antibodies | | |
| Rabbit monoclonal anti-FLAG | MBL | Cat#PM020 |
| Mouse monoclonal anti-β-actin | Huaxingbio | Cat#HX18201 |
| Tubulin-Tracker Red | Beyotime | Cat#C1050 |
| Rabbit monoclonal anti-NF-κB p65 | Cell Signalling Technology | Cat#8242 |
| Rabbit monoclonal anti-Phospho-NF-κB p65 | Cell Signalling Technology | Cat#3033 |
| Rabbit monoclonal anti-p38 | Cell Signalling Technology | Cat#8690 |
| Rabbit monoclonal anti-Phospho-p38 | Cell Signalling Technology | Cat#4511 |
| Rabbit monoclonal anti-IκBα | Cell Signalling Technology | Cat#2859 |
| Rabbit monoclonal anti-GFP | Cell Signalling Technology | Cat#2956 |
| Bacterial and virus strains | | |
| DH5α Chemically Competent Cell | TransGen Biotech | Cat#CD801 |
| BL21 Chemically Competent Cell | TIANGEN | Cat#CB105 |
| Chemicals, peptides, and recombinant proteins | | |
| GST-MyD88 | This paper | N/A (custom-made) |
| MBP-MyD88-WT | This paper | N/A (custom-made) |
| MBP-MyD88-L93P | This paper | N/A (custom-made) |
| MBP-MyD88-L252P | This paper | N/A (custom-made) |
| Lipofectamine 3000 | ThermoFisher | Cat#L3000015 |
| DAPI | Sigma Aldrich | Cat#D9542 |
| DSS | ThermoFisher | Cat# E1483 |
| Passive lysis buffer | Promega | Cat#E1941 |
| Protease inhibitor cocktail | Selleck | Cat#S6598 |
| Critical commercial assays | | |
| Luciferase activity kit | Promega | Cat#E1483 |
| Fast-Mutagenesis system | TransGen Biotech | Cat#FM111 |
| pEasy-Uni Seamless Cloning and Assembly Kit | TransGen Biotech | Cat#CU101 |
| F-actin staining kit | Selleck | Cat#KTC4008 |
| RNAsimple Total RNA Kit | TIANGEN | Cat# DP419 |
| NovoScript Plus All-in-one 1^st^ Strand cDNA synthesis Super mix | Novoprotein | Cat#E047-01B |
| NovoStart SYBR qPCR SuperMix Plus | Novoprotein | Cat#E096-01B |
| Experimental models: Cell lines | | |
| HEK293T | ATCC | Cat#CRL-11268 |
| HeLa | ATCC | Cat#CRM-CCL-2 |
| RAW264.7 | ATCC | Cat#: TIB-71 |
| 293T/17 | National Infrastructure of Cell Line Resource | Cat#1101HUM-PUMC000212 |
| HEK-Blue^TM^ hTLR4 | invivogene | Cat#hkb-htlr4 |
| HEK-Blue^TM^ hTLR5 | invivogene | Cat#hkb-htlr5 |
| Recombinant DNA | | |
| Plasmid: pEGFPN1-MyD88(1-296) | This paper | N/A (custom-made) |
| Plasmid: pmCherry-MyD88(1-296) | This paper | N/A (custom-made) |
| Plasmid: pGEX6P-1-MyD88(20-296) | This paper | N/A (custom-made) |
| Plasmid: pCMV-FLAG-MyD88(1-296) | This paper | N/A (custom-made) |
| Plasmid: pEGFPN1-MyD88(1-117) | This paper | N/A (custom-made) |
| Plasmid: pEGFPN1-MyD88(1-156) | This paper | N/A (custom-made) |
| Plasmid: pEGFPN1-MyD88(20-296) | This paper | N/A (custom-made) |
| Plasmid: pEGFPN1-MyD88(61-157) | This paper | N/A (custom-made) |
| Plasmid: pEGFPN1-MyD88(155-296) | This paper | N/A (custom-made) |
| Plasmid: pEGFPN1-MyD88(20-157) | This paper | N/A (custom-made) |
| Plasmid: pEGFPN1-MyD88(100-296) | This paper | N/A (custom-made) |
| Plasmid: pEGFPN1-MyD88(∆111-154) | This paper | N/A (custom-made) |
| Plasmid: pEGFPN1-MyD88(1-296)-L93P | This paper | N/A (custom-made) |
| Plasmid: pEGFPN1-MyD88(1-296)-R196C | This paper | N/A (custom-made) |
| Plasmid: pEGFPN1-MyD88(1-296)-V204F | This paper | N/A (custom-made) |
| Plasmid: pEGFPN1-MyD88(1-296)-M219T | This paper | N/A (custom-made) |
| Plasmid: pEGFPN1-MyD88(1-296)-L252P | This paper | N/A (custom-made) |
| Plasmid: pEGFPN1-MyD88(155-296)-R196C | This paper | N/A (custom-made) |
| Plasmid: pEGFPN1-MyD88(155-296)-V204F | This paper | N/A (custom-made) |
| Plasmid: pEGFPN1-MyD88(155-296)-M219T | This paper | N/A (custom-made) |
| Plasmid: pEGFPN1-MyD88(155-296)-L252P | This paper | N/A (custom-made) |
| Plasmid: pMBP-MyD88(1-296)-WT | This paper | N/A (custom-made) |
| Plasmid: pMBP-MyD88(1-296)-L93P | This paper | N/A (custom-made) |
| Plasmid: pMBP-MyD88(1-296)-L252P | This paper | N/A (custom-made) |
| Plasmid: pEGFPC1-IRAK4 | This paper | N/A (custom-made) |
| Plasmid: pmCherry-MyD88(1-296)-L93P | This paper | N/A (custom-made) |
| Plasmid: pmCherry-MyD88(1-296)-L252P | This paper | N/A (custom-made) |
| Plasmid: Px458-Myd88-sg | This paper | N/A (custom-made) |
| Plasmid: pUC19-Myd88 template | This paper | N/A (custom-made) |
| Plasmid: pLVX-MyD88-GFP | This paper | N/A (custom-made) |
| Plasmid: pLVX-MyD88 L93P-GFP | This paper | N/A (custom-made) |
| Plasmid: pLVX-MyD88 L252P-GFP | This paper | N/A (custom-made) |
| Plasmid: pEGFP-N1 | Clontech | Cat#6085-1 |
| Software and algorithms | | |
| ImageJ | NIH | https://imagej.net/imagej-wiki-static/Fiji |
| GraphPad Prism 8 | GraphPad Software Inc | https://www.graphpad.com/scientific-software/prism/ |
| PyMOL | Molecular Graphics System, Schrodinger, LLC | https://pymol.org/2/ |
| AMBER18 | Case et al., 2018 | http://ambermd.org/ |
| VMD | Humphrey et al., 1996; Best et al., 2012 | https://www.ks.uiuc.edu/Research/vmd/ |
| Matplotlib | Hunter, 2007 | https://matplotlib.org/ |
